# Notes and News

**Published:** 1887-12-17

**Authors:** 


### Notes and News.

Collected and Communicated.

It is gratifying to be able to announce that the Hospital Saturday collections at Wolverhampton showed a considerable increase over those of last year. The sums collected amounted to £1,250. A special collection was made on behalf of the Eye Hospital, which realised £228.

A public meeting of the medical profession was held at the Society of Arts, under the presidency of Sir T. Spencer Wells, to consider the report of the Medical Attendance Organisation Committee, which recommended a comprehensive scheme of medical attendance for the industrial classes in the metropolis, on a self-supporting basis, involving the creation of dispensaries in each district; these dispensaries to form a union with the hospital. The suggestion provoked a good deal of unfavourable comment, but a resolution approving of it was finally passed by a large majority.

Mr. F. Armitage writes in *The Queen*: "A most benevolent and useful scheme has been set on foot for the benefit of tired hospital nurses, which deserves passing notice. Sister Philippa, of King's College Hospital, finding by experience that, when sadly needing a few weeks' rest and holiday, many of these devoted women had neither friends nor relations whom they could visit, started the idea of getting ladies in the country to invite one or more nurses during the year to stay in their family. By this means a more complete time of rest, relaxation, and change is obtained than by a far longer stay in some institution. The plan has been tried for one year, and has proved so great a boon to the nurses in many of our large city hospitals, that Sister Philippa is anxious to extend it, by getting very many more ladies to offer a fortnight's change to those who need it. One feels that in large houses such an addition to the household would make no difference; and to others, who have small means but large hearts full of charity and love towards those whose lives are devoted to the sick and suffering, the admission of one of the lady nurses to their own circle for a short time is no inconvenience. It must be remembered that to get quite away from the sad routine of sick nursing into fresh scenes and new surroundings is the most refreshing and invigorating stimulant to renewed energy for work. The sister who is working this "Rest for Nurses" is a practical woman, carefully arranging the details, and sending nurses to those houses where the circumstances of hostess and guest will best assimilate. She has carried it out so far without any appeal for monetary support, and her wish is to develop it to a much larger extent, and she will send further particulars to anyone applying to King's College Hospital."

The meeting of the constituents of the Hospital Sunday Fund, on Monday at the Guildhall, was well attended and sympathetic. The Rev. W. H. Barlow, B.D., Vicar of Islington, bore eloquent testimony to the value of the Hospitals Week, which had increased the collections at his church fifty per cent. He expressed a hope that the public meetings in the week preceding Hospital Sunday would continue, as they excited much interest and did great good. Dr. Barlow bore independent and valuable testimony to the service rendered by The Hospital to the Hospital Sunday Fund in co-operating with the council to the extent of issuing special supplements for the information and assistance of speakers and preachers. Another noteworthy feature was the presence, for the first time we believe, of ladies, who attended as the representatives of congregations in various parts of London. This is a good sign, and if the ladies in every parish and congregation will respond to the appeal made to them by Sir Andrew Clark and organise a Hospital Society in connexion with every religious organisation throughout London, the sick poor will be better cared for and the funds of the hospitals will greatly increase.

Mr. H. Nelson Hardy, F.R.C.S., the winner of the Sturge Prize for the best essay on hospital administration, moved an amendment which the Lord Mayor ruled to be out of order, and which was subsequently withdrawn. Mr. Hardy contended that South-East London contributed one-fourth of the funds and received back only one-twelfth in grants to charities situated within the district. He was under the erroneous impression that the Seamen's Hospital did not admit local cases, whereas upwards of two thousand such patients are there treated there every year. He desired that Guy's Hospital should take a substantial grant from the fund, and his amendment aimed at an alteration of the constitution which would permit Guy's Hospital to receive a grant on a different basis to that laid down for the voluntary hospitals. Sir Sydney Waterlow, and subsequently Mr. Alfred Cohen (a governor of Guy's Hospital), urged the withdrawal of the amendment on the ground that Guy's Hospital would be eligible to participate in the fund so soon as the treasurer presented the accounts for the three last years, and conformed to the conditions accepted by all the other participating charities. Mr. Cohen added that the financial condition of Guy's was improving rapidly, that the accounts for the last year, at any rate, had been put into perfect order, and that there was some reason to believe that every bed in Guy's Hospital would soon be occupied, as the finances were so improved as to permit of this being done without much further delay.

Although we desire to see Guy's Hospital flourish to the utmost, we agree with the *Standard* in viewing the above proposals with disfavour, because "if each district is to keep watch upon its own contributions to the fund, and find a grievance if every farthing does not come back to it in the form of doles, there is no use in having a general fund at all. It is of the essence of such a fund that one district should support another. Otherwise each locality had better be left to its own efforts." We have reason to know that the views propounded by the mover of the amendment are not those of a large majority of people in South-East London, who recognise the inhumanity of sending all their cases over miles and miles of rough stones to seek admission to Guy's Hospital. The Miller Memorial Hospital, and the Blackheath, Beckenham, Bromley, and Eltham Cottage Hospitals, more than one of which are about to be extended, are proof positive that the residents in South-East London are beginning to recognise the duty of providing for their own sick poor within a reasonable distance of their homes. So soon as South-East London provides hospital accommodation to an extent equal to that in other districts, so soon will the grants made to the institutions working amongst its poor equal those received by the more numerous and larger charities elsewhere.

The following vacancies are announced this week, the amount of salary in each case being given after the name of the institution :—

**Resident Medical Officers.**

Ashton-under-Lyne Infirmary. House Surgeon.—£80.
Pontefract General Dispensary. House Surgeon.—£150, with rooms.
North-Eastern Hospital for Children, Hackney Road. Junior House Surgeon.—£50.
Birmingham General Hospital. Assistant House Surgeon.
Royal Albert Hospital, Devonport. Assistant House Surgeon.
Hospital for Consumption, Brompton. Resident Clinical Assistant.
Royal Westminster Ophthalmic Hospital, King William Street. House Surgeon.

**Non-resident Medical Officers.**

Samaritan Free Hospital for Women and Children. Physician.
St. George's Hospital. Assistant Physician.
St. George's Hospital. Assistant Surgeon.
St. George's Hospital. Physician.
St. George's Hospital. Surgeon.

**Matron.**

Batley and District Cottage Hospital (20 beds). Matron, hospital trained, with knowledge of dispensary preferred.—£50.

**Nurses.**

West Derby Union. Nurse at the Infirmary, Mill Road, Liverpool.—£18 for first year, £20 second, and £1 annually thereafter till £25 is reached.
Mr. Wilson's Nursing Institute, Wimpole Street, W. Nurses wanted for fever cases, age 25 to 36.—Salary £50.
Ross Memorial Hospital, Dingwall, N.B. Fever Nurse.
Tyrrell Hospital, Ilfracombe. Assistant Nurse.—£24, uniform and laundress.

---

During the last seven years, annual series of "Health Lectures for the People" have been delivered in Edinburgh by professors of the University, lecturers at the extramural schools, and medical men of repute. The lectures are free, though anyone who so chooses may contribute a small sum—from a halfpenny upwards—towards the expense of getting them up. The lectures are subsequently printed in pamphlet form and sold at a penny each, and on the conclusion of the series the various numbers are combined in a small paper covered volume, published by Messrs. Macniven and Wallace, Edinburgh, at the price of one shilling. For the interest of our readers, we append a few quotations from the most recent series of these addresses.

William Stirling, M.D., D.Sc., Professor of Physiology in the Owens College (Victoria University), Manchester, says, in a lecture on "Tear and Wear": "The heart beats for a certain time and rests for a certain time. As a matter of fact, the working heart spends two-fifths of its time in action, and three-fifths of its time in rest or a period of repose. An increase of temperature, no matter how it is caused, leads to an increased waste, leads to an increased tear and wear of all the tissues in the body. The heart in fever beats more rapidly, partly because the temperature is higher; and you will see, therefore, that in fever the heart is subjected to an excessive strain. Hence one of the reasons why prolonged fever produces so serious an effect upon the heart. The heart having so short a time for repairing the waste that goes on in it, is apt to break down under the strain, and, in fact, in many cases undergoes an actual change in its structure."

---

J. Cossar Ewart, M.D., Professor of Natural History in the University of Edinburgh, lecturing on "The Rôle of Bacteria in Health and Disease," says: "If bacteria are heated considerably they are utterly destroyed. Then the air we can purify to a very large extent. As the result of numerous experiments, we have learned that the simplest way of purifying the air is to saturate it with a mixture of steam and heat. Sulphurous acid is often used to purify rooms, but it is practically useless. Chlorine is more useful, but it does not penetrate sufficiently to kill all the organisms. If, however, we saturate the atmosphere of any room with a mixture of steam and hot air, almost every organism is destroyed. In the same way it is possible to purify clothing and furniture. If we take a thick roll of blankets, into the centre of which spores have been previously introduced, and treat them with a mixture of hot air and steam, the steam penetrates in a short time through the blankets and kills the bacteria."

---

Dr. Stevenson Macadam, F.R.S.E., Lecturer on Chemistry at the Surgeons' Hall, Edinburgh, addressing his audience on "Soap, Soda, and other Cleansing Materials," says: "Accept and use all cleansing materials—soap and soda—as aids to and not substitutes for scrubbing and rubbing. Never shirk what is popularly known as "elbow grease." Use soap equally in moderation for personal washing, and exercise the hands freely, so that the skin may retain its natural healthy secretive power, which is so conducive to good health."

---

G. A. Gibson, M.D., speaking of headaches, gives a brief but clear description of the anatomy of the brain, and goes on to say, in describing the various kinds of headaches: "The rheumatic headache, which is vastly common in this country, has its position somewhat widely extended over a large area of the brow, the temples, or the back of the head. It is a muscular pain of a dull, aching character, and the painful parts are always tender to pressure and worse at night. It is often induced by a damp atmosphere or a draught of cold air. For its relief is required the treatment of the rheumatic tendency, with careful regulation of diet and clothing. Cold water in this condition is only to be applied to the head with caution. In the state known as 'plethora' there are frequent headaches, attended by fulness of the head, sleeplessness, giddiness, singing in the ears, and weakness of sight. The pain is always increased by any stimulus, such as light or sound; even thinking adds to the suffering. Saline aperients, with low diet and abstinence from every kind of excitement, are specially called for if such an attack has come on; while careful regulation of food, drink, work, and rest must be insisted on as means of prevention. Sometimes in this condition there is bleeding from the nose; it is not to be stopped.

---

John Smith. M.D., LL.D., F.R.S., dental surgeon to the Queen in Scotland, lecturing on "The Physiology and Functions of the Teeth," says: "The number of diseases alleged to be connected with 'teething,' or cutting the milk teeth, is probably exaggerated; and again, irregularity of the second set appears to be due in some measure to the absence of any reserve spaces in the dental series, such as occur in the lower animals, as well as to occasional arrest in the growth of the upper and lower jaws. The prevention of decay is to be sought in maintaining the general health, and, as a local measure, in cleanliness of the teeth by removing rom them decomposing agencies of a destructive nature."

---

A correspondent of the *Nottingham Daily Express* writes to complain of the existence of a fever hospital—he calls it a den—in Woodborough Road. He says "it is monstrous that a respectable part of the community should be annoyed with the sight of these miserable fever sheds." He also resents the air being "poisoned with the fumes of sulphur arising from the disinfecting ward." We do not pretend that a fever hospital is in itself a pleasing sight, but while there is fever in a town—and this gentleman offers no suggestion for getting rid of *that*—there must be a hospital to receive those who suffer from it, and it is hardly possible to hide it entirely from the public gaze. We take this opportunity of explaining to the irate inhabitant of Nottingham, whose complaint we have here noted, that though it is unfortunate that the fumes of sulphur should come "between the wind and his nobility," the said fumes do not "poison" the air—that is, do not make it dangerous to human life; indeed, many people consider them rather wholesome than the reverse.

A bazaar, in aid of the local hospital was held at Gravesend on the 1st inst., when the stallholders and their young lady assistants were appropriately dressed in the costume of hospital nurses.

The annual contract for the supply of Edwards' preserved potato to her Majesty's Navy, 145,000 lbs., has again been taken by Messrs. Frederick King and Co. (Limited), of Belfast and London, who have supplied the Admiralty for over forty years. Their Edwards' Desiccated Soup is well known in the home market.

We regret to note that the results of the Hospital Sunday collections at Richmond show a falling off as compared with previous years. No doubt the whilom contributors have had other calls upon their generosity, but we hope that they were sure, before they withheld or lessened their contributions to the Hospital Sunday Fund, that they meant to expend their money on as good an object. They could hardly find a better.

The following notice appeared in the *St. James's Gazette* of the 9th inst. : "A British Nurses' Association.—A representative gathering of the matrons of the metropolitan hospitals and infirmaries, and other members of the nursing profession was held on Wednesday evening, at the residence of Mrs. Bedford Fenwick, to discuss the advisability of forming an association for the mutual help of all women engaged in nursing the sick, and for the advancement generally of their professional usefulness. It was unanimously agreed that a 'British Nurses' Association' should be formed. Bye-laws were passed, a general council constituted, and an executive committee appointed." Two remarks may be made in regard to this *communiqué*. First, the modesty of the representative gathering in refraining from giving the names of those present; and secondly, the despatch with which bye-laws, regulations, councils, and committee were formulated, passed, and constituted. No mention is made of the fact that nurses were trained, registered, and granted pensions on this eventful evening, but possibly considerations of space prevented the writer from making this further announcement. It will be seen from a paragraph elsewhere that those who have heretofore been rightly regarded as the leaders and representatives of the matrons of the metropolitan hospitals, who hold certificates, and are therefore entitled to speak with authority, have joined the Registration Committee of the Hospitals Association.

The council of The Hospitals Association decided on Tuesday to appoint a joint committee of ladies and gentlemen to receive applications from nurses desiring to be registered, and to conduct the business connected therewith. The Registration Committee will consist of fifteen members, with power to add to their number, those already appointed being: Mrs. Wardroper, Miss Lückes (or a representative of the London Hospital), Miss Vincent (Marylebone Infirmary), Mrs. Bluett (Fitzroy House), Miss Styring (Paddington Infirmary), Miss L. E. Baxter (New Hospital for Women), Dr. Steele (Guy's), Mr. John Croft, F.R.C.S. (senior surgeon St. Thomas's), Dr. C. Theodore Williams (physician to the Brompton Consumption Hospital), Dr. Rayner (Hanwell Asylum), Colonel Montefiore, Mr. P. J. Michelli (secretary Seaman's Hospital), and Dr. Stevenson (St. Bartholomew's). Miss Jones, of Guy's, has also been invited to join this committee. It was further determined to refer to this committee the question of who should be admitted to the register, and whether any nurse should be eligible for registration unless she has worked for at least one year on the staff of a hospital or infirmary, and two years in an institution or institutions co-operating more or less with a hospital. Any nurses who desire to be registered or who wish for information should communicate with the Secretary, The Hospitals Association, Norfolk House, Norfolk Street, W.C.

Ten thousand pounds is the sum distributed among the London charities as the results of the efforts of the Hospital Saturday Fund.

The latest proposal in connexion with the Colchester Hospital is to appoint a physician to the institution, and to give him a stipend of £200 a-year. The suggestion appears to be very unpopular at Colchester, which is not surprising. The Medical Board are said to be responsible for it, and the local Press speaks in no measured terms of their "temper," "fancies," and "whims." We are not familiar with the *personnel* of the profession at Colchester, but it would seem that among so many practitioners there should be at least one or more who is worthy to hold the position of honorary physician to the hospital. If there be not, it is very doubtful whether such a paltry stipend as £200 a-year would induce anyone superior to the local practitioners to accept the office.

A very pretty cottage hospital has just been opened at Ruabon. It has been built by Sir Watkin Williams Wynn as a memorial of his late uncle, the sixth baronet, who, from the time the hospital movement was initiated in the district, eighteen years ago, on the occasion of a serious accident at the Green Pit, showed himself most anxious to promote it. It was to his and Lady Wynn's energy and thoughtfulness that Ruabon owed its original hospital accommodation, and before his death he had selected the site for the present building. In a district where coal mining is the chief industry, a hospital is a matter of necessity, and Sir Watkin Wynn is to be congratulated on having so well done his duty to the place in which he lives. The hospital, which is an exceedingly pretty building, was designed by Mr. W. H. Spaull, of Oswestry.

H.R.H. the Prince of Wales did a great service to the artisans of England by visiting the East-end of London on Saturday last and opening the Apprentices' Exhibition at the People's Palace. This excellent movement on the part of Sir Edmund Hay Currie and the Trustees of the Beaumont Trust promises to do much good in connexion with the technical schools which have been founded there, and is, as the Prince pointed out in his admirable reply to the address presented him, the beginning of a movement which must become general, and by which the British workman will be educated to keep step with the march of modern invention. "The English artisan must no longer be content to be as good as his father, he must by diligent study render himself better than his father; there will be no need then to fear foreign competition"—sound advice which may well be taken to heart by all. Sir Lyon Playfair made an able and impressive speech, thanking his Royal Highness for the assistance he was rendering the cause of the working man. He hoped that the time was not far distant when the workmen of a factory would no longer be reckoned only as hands, but that the importance of their heads would be acknowledged as well.

The reception of the Prince was most hearty, and after he had witnessed a miscellaneous display in the gymnasium, he was conducted through the exhibition, comprising all branches of art and manufacture, by Sir Edmund Currie; to whom, in spite of his endeavour to keep himself in the background, it is recognised by all that the success of the Palace is chiefly due. The apprentices of London, in the widest sense of the term apprentice, many of whom are pupils of the Polytechnic School in Regent Street, have supplied altogether 897 exhibits, of which many are of a highly artistic and scientific nature, and full of the greatest promise. The Queen's Hall, in which the addresses were made, was crowded with an enthusiastic, representative, and attentive audience, who had been admitted by ticket. It is to be hoped that many philanthropic men will pay a visit to this exhibition, and be encouraged thereby to further the important cause of technical education by every means in their power.

